# Gamma-secretase-dependent signaling of receptor tyrosine kinases

**DOI:** 10.1038/s41388-018-0465-z

**Published:** 2018-08-30

**Authors:** Johannes A.M. Merilahti, Klaus Elenius

**Affiliations:** 1 Institute of Biomedicine, University of Turku, 20520 Turku, Finland; 2 Medicity Research Laboratory, University of Turku, 20520 Turku, Finland; 3 Turku Doctoral Programme of Molecular Medicine, University of Turku, 20520 Turku, Finland; 4 Department of Oncology, Turku University Hospital, 20520 Turku, Finland

**Keywords:** Growth factor signalling, Cancer

## Abstract

Human genome harbors 55 receptor tyrosine kinases (RTK). At least half of the RTKs have been reported to be cleaved by gamma-secretase-mediated regulated intramembrane proteolysis. The two-step process involves releasing the RTK ectodomain to the extracellular space by proteolytic cleavage called shedding, followed by cleavage in the RTK transmembrane domain by the gamma-secretase complex resulting in release of a soluble RTK intracellular domain. This intracellular domain, including the tyrosine kinase domain, can in turn translocate to various cellular compartments, such as the nucleus or proteasome. The soluble intracellular domain may interact with transcriptional regulators and other proteins to induce specific effects on cell survival, proliferation, and differentiation, establishing an additional signaling mode for the cleavable RTKs. On the other hand, the same process can facilitate RTK turnover and proteasomal degradation. In this review we focus on the regulation of RTK shedding and gamma-secretase cleavage, as well as signaling promoted by the soluble RTK ICDs. In addition, therapeutic implications of increased knowledge on RTK cleavage on cancer drug development are discussed.

## Introduction

Human genome harbors genes encoding 55 receptor tyrosine kinases (RTK) [1]. RTKs can signal through traditional, canonical pathways involving other kinases and lipid messengers but can also signal through a noncanonical pathway involving the proteolytic release of intracellular domain fragments (ICD), which translocate to various cellular compartments [2–4].

Full-length RTKs and RTK fragments have been observed in the intracellular cell compartments such as nucleus and mitochondria [4]. Active RTK fragments can be generated by proteolytic cleavage resulting in the release of soluble RTK ICDs from the membrane. These fragments can be generated by various mechanisms in response to various stimuli, including caspase cleavage and gamma-secretase-dependent pathways. In this review we will focus on gamma-secretase-dependent cleavage pathways. Other mechanisms of RTK processing have been recently reviewed elsewhere [5].

## Generation of soluble ICD by ectodomain shedding and gamma-secretase cleavage

ERBB4 was recognized as the first RTK cleavable by gamma-secretase almost 20 years ago [6]. Since then the amount of RTKs identified as substrates have gradually increased to a point that approximately half of the RTKs have been reported to be cleaved by gamma-secretase (Table 1). The prevalence of this phenomenon among RTKs indicates that cleavage-associated and -generated signaling can play a major role in RTK signaling.

**Table 1:** RTKs reported to be cleaved by gamma-secretase

RTK	Sheddase	Reference for sheddase	Reference for gamma-secretase cleavage
AXL	ADAM10/ADAM17	[108]	[110]
CSF1R	ADAM17	[111]	[96]
EphA2	MT-MMP	[112]	[17]
EphA4	ADAM19	[113]	[114]
EphA5			[17]
EphA7			[17]
EPHB2	ADAM10	[29]	[29]
EPHB3			[17]
EPHB4	ADAM8	[115]	[17]
EPHB6			[17]
ERBB4	ADAM17	[80]	[6]
FGFR3	Metalloprotease	[30]	[30]
FGFR4			[17]
IGF1R			[97]
INSR	ADAM17	[116]	[116]
MER	ADAM17	[117]	[17]
MET	ADAM10/ADAM17	[98, 118]	[98]
MUSK			[17]
PTK7	ADAM17	[66]	[66]
RYK	Metalloprotease	[119]	[31]
TIE1	metalloprotease	[99]	[99]
TRKA	Metalloprotease	[120]	[17]
TRKB	Metalloprotease	[93]	[93]
TYRO3			[17]
VEGFR1	ADAM8/ADAM10/ADAM17	[115, 121]	[71]
VEGFR2	ADAM17	[122]	[101]
VEGFR3			[17]

In total, 27 human RTKs (out of the 55 encoded by genome) have been reported to be cleaved by gamma-secretase. Proteases indicated to be responsible of ectodomain shedding are also listed, when known

Most of the work addressing the molecular mechanisms by which the gamma-secretase complex recognizes and interacts with its substrates has been carried out with Alzheimer’s disease-related amyloid precursor protein (APP) and to some extent with the notch receptors. Only relatively little research has been conducted on the intracellular cleavage of RTKs and, so far, practically no work has addressed aspects of substrate recognition, substrate binding, and actual cleavage catalysis in the context of RTKs. Thus, it is plausible that gamma-secretase-mediated cleavage is regulated by mechanisms resembling those exploited by both APP and notch, but this remains to be confirmed.

**Fig. 1:**
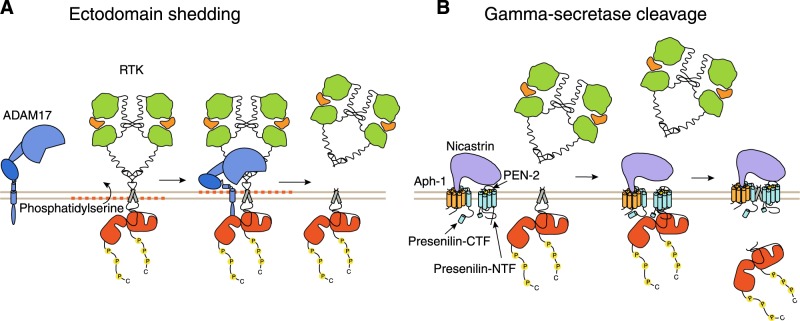
Regulated intramembrane proteolysis of RTKs. **a** RTK ectodomains are shed by cleavage at the extracellular juxtamembrane domains by sheddases, such as ADAM17 or ADAM10. Ligand activation of RTK may result in exposure of phosphatidylserines on the cell surface resulting in a conformational change in ADAM17 facilitating access to the RTK cleavage site. **b** After shedding of the ectodomain, gamma-secretase complex, consisting of APH-1, nicastrin, PEN-2, and presenilin, gains access to membrane-bound RTK fragment. Nicastrin can now interact with the short RTK ectodomain. RTK binding to the gamma-secretase complex induces further conformational changes in the gamma-secretase subunits that result in substrate translocation to the active site. Cleavage of the RTK substrate in transmembrane domain results in the release of the soluble ICD into the cell cytoplasm. RTK receptor tyrosine kinase, APH-1 anterior pharynx defective-1, PEN-2 presenilin enchancer-2, ICD intracellular domain fragment

The formation of a soluble ICD requires the full-length receptor to be cleaved by two sequential proteolytic cleavage events in a process called regulated intramembrane proteolysis (RIP; Fig. 1) [7]. At first, the extracellular ectodomain of the receptor is cleaved and released into extracellular space by a sheddase. Alpha-secretases such as matrix metalloproteinases (MMP) and members of a disintegrin and metalloproteinase (ADAM) family, and beta-secretases such as aspartic proteases, promote the ectodomain shedding of gamma-secretase substrates by proteolytic cleavage adjacent to the plasma membrane in the extracellular juxtamembrane part of the receptor (Fig. 1a). ADAM10 and ADAM17 (tumor necrosis factor alpha-converting enzyme; TACE) are responsible for most of the RTK ectodomain shedding. Some RTKs are subject to cleavage by both of these sheddases (Table 1).

The ectodomain shedding is followed by the secondary cleavage event at the receptor transmembrane domain by gamma-secretase intramembrane protease complex, releasing an intracellular protein fragment (Fig. 1b). Indeed, only transmembrane proteins with ectodomains within 12−35 amino acids in length (the length typically generated by shedding) function as substrates for gamma-secretase [8].

Gamma-secretase protein complex is composed of four subunits: presenilin, which is proteolytically cleaved to N-terminal and C-terminal fragments, presenilin enchancer-2 (PEN-2), anterior pharynx defective-1 (APH-1), and nicastrin [9]. Human proteome harbors two isoforms for presenilin and three for APH-1 creating multiple possible combinations for composition of the protein complex. Nicastrin binds to free N-termini of substrates and excludes substrates with too large free N-termini [10, 11], explaining the requirement for prior ectodomain shortening by sheddases for the correct assembly of gamma-secretase complex on substrates such as RTKs. Presenilin harbors the enzymatic activity of gamma-secretase complex and cleaves RTKs among other type I single transmembrane proteins [12]. The actual roles of other subunits are still unclear. The cleavage results in the release of a soluble ICD from the cell membrane into the cell cytoplasm (Fig. 1b).

The requirement for substantial conformational changes in the gamma-secretase complex during the substrate binding and catalysis of the cleavage have been reported [11]. The substrates bind to gamma-secretase at a substrate binding site, from which they are then transferred to a docking site in the vicinity or overlapping the catalytic site. It has been demonstrated that substrate movement out from the binding site to catalytic site is required for cleavage [11].

## RTK recognition by gamma-secretase

A conserved structural motif in the transmembrane domain of substrates is probably needed for the recognition by gamma-secretase complex. So far, no conserved amino acid consensus sequence has been identified serving either as the substrate recognition sequence or as the actual substrate cleavage site. The recognition of substrates is thought to be based on a combination of the length of the remaining ectodomain after shedding [8], the strength of interaction between the substrate transmembrane domain and the gamma-secretase complex [10] and subcellular localization of the substrate in relation to gamma-secretase activity [13, 14]. Interestingly, gamma-secretase complex may also cleave specific substrates directly with no need for shedding [15, 16], in cases where the ectodomains are naturally short enough to circumvent the size exclusion step provided by the nicastrin subunit for substrate recognition [10].

While most of the substrate interactions with gamma-secretase complex have been indicated to take place within N- and C-terminal presenilin sequences, interactions of the substrates with PEN-2 and nicastrin subunits have also been observed [11]. Nicastrin and PEN-2 have been shown to interact with amino acid residues close to the N-terminus of the substrate. Recent data indicate that nicastrin acts as a gatekeeper for substrates and obstructs the entrance of large ectodomain containing substrates [10]. In the proposed model of gamma-secretase cleavage by Fukumori and Steiner, substrates interact with nicastrin and PEN-2 when substrates are introduced to gamma-secretase complex [11]. However, a complete picture of the structural elements guiding RTK recognition by the gamma-secretase complex remains to be elucidated.

## Regulation of ICD formation

Gamma-secretase has over 100 identified substrates [12, 17, 18] but known substrates represent only a minor fraction from the potential ones in the human proteome, which includes about 2500 single span membrane proteins [19, 20]. In the same manner as any proteolytic process intramembrane proteolysis requires substrate identification, cleavage of the scissile bond at the active site of the protease, and finally the release of a product [21]. However, ectodomain shedding and subsequent gamma-secretase cleavage are irreversible procedures unlike many other signaling mechanisms and hence must be tightly regulated.

The main factors that regulate RTK RIP are (i) ectodomain shedding that is required before gamma-secretase cleavage and is thus critical for the initial selection of substrates, and (ii) subcellular localization of substrates and gamma-secretase complex in relation to each other.

### Regulation of RTK RIP by sheddases

While gamma-secretase cleavage of cell surface proteins could be considered a relatively passive event taking place almost automatically after exposure of the substrate by prior ectodomain shedding, it is not promiscuous. In agreement, not all RTKs that have been reported to be shed undergo cleavage by gamma-secretase. RTKs such as ERBB2 and TIE2 have been found to undergo ectodomain shedding, but no indications for gamma-secretase cleavage has been reported so far [22, 23]. This could be explained by that further cleavage by gamma-secretase proceeds only in specific molecular or subcellular contexts [10, 11, 13, 14, 24] or that the half-life of the released ICD is so short that it is not easily observed without treating cells with reagents that increase the cleavage or inhibit the degradation of ICDs [25].

In any case, as shedding is typically a prerequisite for subsequent processing, RTK cleavage by gamma-secretase is in principle subject to control by any circumstances that affect the expression or activation of the sheddase. For example, it has been shown that ERBB4 ectodomain is present in the serum of the breast cancer patients at higher concentrations than in the serum of healthy individuals, and that the enhanced shedding associates with more ADAM17 protein present in breast cancer tissue as compared to paired samples representing histologically normal adjacent breast tissue [26]. Upregulation of ADAM expression has also been reported in other cancer types, and number of effectors of ADAM gene expression have been defined, including cytokines and growth factors [27, 28].

While basal RTK cleavage has been reported, quite often activation by ligand is needed for RTKs to be cleaved by gamma-secretase. For example, ligand-induced cleavage has been observed for CSF1R, EPHB2, ERBB4, FGFR3, RYK, VEGFR1, and VEGFR2 [6, 29–34]. The activation of RTK cleavage by ligands may be due to activation of downstream signaling kinases that result in activation of shedding by phosphorylation of the cytoplasmic tail of ADAM17 and/or ADAM10. These mechanisms include the activation of PLK2, MAPKs, and PKC (reviewed in refs. [35, 36]).

In addition to ligand activation, activation of other cellular receptors and changes in ion concentrations across the cellular membranes have been associated with induction of gamma-secretase cleavage. Examples include cleavage of CSF1R, EPHB2, and ERBB4 RTKs [26, 29, 37]. The likely explanation is that these molecules that promote activation of cleavage increase the activity or the amount of sheddases promoting the shedding, and as such generate substrates for subsequent gamma-secretase cleavage.

It has further been indicated that shedding of ectodomains requires phosphatidylserine exposure at the outer layer of the cell surface that interacts with ADAM17 membrane-proximal-domain leading to a conformational change in the ADAM17 ectodomain enabling its catalytic site to interact with cleavage sites of its transmembrane substrates [38, 39] (Fig. 1a). Overall, it has been proposed that to achieve shedding activity, ADAM17 has to be activated while ADAM10 is more constitutively active (reviewed in ref. [36]).

The regulation of gamma-secretase cleavage at the level of shedding may also be modified by *O*-glycosylation of the substrates [40] Sheddases cleave substrates at membrane proximal extracellular juxtamembrane domain, which is also where *O*-glycosylation frequently takes place. Glycosylation has been found to predominantly reduce ADAM17-mediated shedding, with some reports indicating alterations in cleavage sites after glycosylation as well [40]. The sites of *O*-glycosylation are controlled by only one or few GalNac-transferase isoforms indicating that shedding could be regulated to some extent by expression of specific GalNac-transferase isoforms [40]. Interestingly, an ERBB4 mutation Q646C that creates a constitutively active ERBB4 [41] and is situated at one of the proposed ADAM17 cleavage sites [40] was shown to decrease the efficiency of site-specific *O*-glycosylation [40]. Hence, it is conceivable that *O*-linked glycosylation of RTKs influences the intracellular cleavage and signaling by reducing ectodomain shedding.

### Subcellular localization in regulation of RTK RIP

It has been reported that most of the mature ADAM17 is localized intracellularly as a result of constitutive internalization, with only a small fraction residing on the cell surface [42]. Furthermore, most of the intracellular ADAM17 is in inactive form in endoplasmic reticulum (ER) and its maturation takes place in the Golgi apparatus. The trafficking from ER to Golgi is dependent on iRhoms 1 and 2 [43–46]. Furthermore, ADAM17 substrate selectivity, shedding activity, and cell surface stability have been indicated to be regulated by iRhoms [47–49]. In addition, sorting protein PACS-2 is involved in trafficking and sustaining cell surface activity of ADAM17 [50]. The cell surface localized ADAM17 has been observed to be regulated by tissue inhibitor of metalloproteinases 3 (TIMP3), which binds to catalytic domain of ADAM17 [51]. ADAM10 can be regulated by TIMP1 as well as by TIMP3 [52]. In addition, integrin beta1 interaction with ADAM17 has been associated with inhibition of cell surface ADAM17 [53, 54].

Chen et al. showed that ADAM10 and gamma-secretase complex reside in the same multiprotease complex [55]. While the study focused on ADAM10, it is plausible that other sheddases reside in their own multiprotease complexes with gamma secretase. Indeed, it was demonstrated that other sheddases such as ADAM17 did not immunoprecipitate with ADAM10 in contrast to gamma-secretase complex, which did immunoprecipitate with ADAM10 [55]. Both sheddases and gamma-secretase residing in same complexes can in principle result in optimized processivity of the cleavage event by promoting efficient ICD release after shedding. However, further research is needed to illustrate the composition of the protease complexes used for each RTK.

Different gamma-secretase complexes containing either presenilin-1 or presenilin-2 have been reported to direct their gamma-secretase complexes to distinct subcellular localizations [13, 14]. Presenilin-2-containing gamma-secretases are directed to late endosomes/lysosomes [14] while presenilin-1-containing complexes are distributed more diffusely in the cell membranes. It has also been shown that composition of the lipid membrane affects the localization and activity of gamma-secretase as well as its substrates. Presenilin-1-containing gamma-secretase complexes have been shown to localize to lipid rafts and the composition of lipids to affect the activity of the complex [56–59]. In addition, it has been observed that gamma-secretase cleavage is quite a slow process compared to similar cleavage events executed by soluble enzymes [60]. It was demonstrated that the strength of substrate/enzyme interaction can regulate the overall reaction rate of gamma-secretase, and that rate of proteolysis is influenced by substrate binding and processing. Taken together, these observations indicate that proper spatial distribution and compartmentalization of gamma-secretase and substrates is needed for appropriate cleavage process.

## Intracellular fate of cleaved ICD

Cleaved ICDs of RTKs have been detected in various cellular subcompartments. Also in this respect, the most-studied RTK is ERBB4. Soluble ERBB4 ICD, as detected by overexpressing constructs encoding solely the ICD domain, or by analyzing endogenously produced ICD fragments using subcellular fractionation analyses and chemical inhibitors of nuclear export, has been detected in nucleus by several models both in vitro and in vivo [6, 25, 61–63]. Cell types that naturally produce ERBB4 ICD in their nuclei include mammary epithelial cells and other breast cancer cells. Interestingly, it has been reported that more nuclearly localized ERBB4 is detected in breast cancer tissue than in normal breast [64] and that nuclear ERBB4 localization associates with worse prognosis than cell surface localization in breast cancer [65].

In addition to ERBB4, soluble ICDs of endogenously expressed AXL, RYK, and TRKA have been reported to translocate to the cell nucleus while those of FGFR3 and PTK7 have only been observed in nucleus when overexpressed [17, 30, 31, 66–68]. The ICDs of AXL, FGFR3, and PTK7 have been observed in the nucleus in vitro [17, 30, 66, 67], RYK in an in vivo mouse model [31], and TRKA in immunohistochemical analysis of human liver tissue samples [68].

Soluble ERBB4 ICD has also been reported to translocate into the mitochondria [69, 70] while the ICDs of EPHB2 and VEGFR1 remain in the cytosol of the cell [71, 72]. The mechanisms governing the translocation of the released ICDs into various cellular organelles after the cleavage remain largely unknown. However, modification of ERBB4 ICD by the small ubiquitin-like modifier (SUMO) has been shown to promote nuclear localization by a mechanism probably involving enhanced kinase activity and inhibition of nuclear export of ERBB4 ICD from the nucleus back to the cytosol [73, 74].

As discussed below, the soluble ICD of several RTKs may also rapidly translocate to proteasomes for degradation.

## Cellular functions regulated by cleaved RTK ICDs

**Fig. 2:**
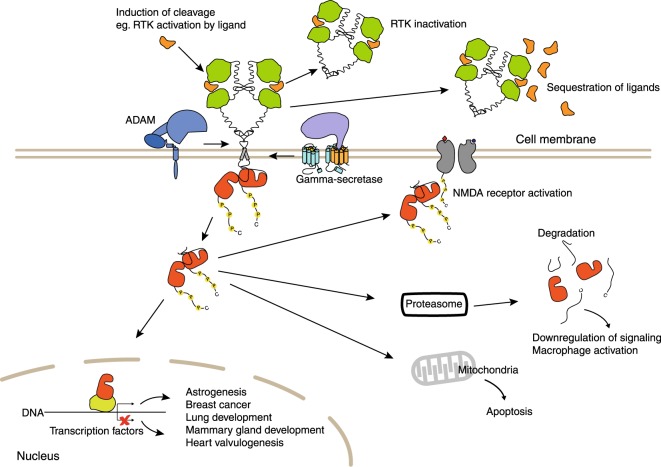
Functional significance of RTK fragments generated by RIP. The reported functions associated with nuclear localization of ICDs include roles in neural development, mammary gland development, and lung development as well as with breast cancer. Mitochondrial localization has been associated with regulation of apoptosis. Cytoplasmic ICD localization allows activation of other receptors, such as NMDA. Downregulation of RTK signaling has also been associated with rapid localization of ICDs to proteasome after gamma-secretase cleavage. The release of shed ectodomains may also contribute to downregulation of RTK signaling by sequestering ligands. RTK receptor tyrosine kinase, RIP regulated intramembrane proteolysis, ICD intracellular domain fragment, NMDA *N*-methyl-d-aspartate

After the release from the membrane, the RTK ICDs can act as signaling molecules with various functions (Fig. 2). The ICDs, again mostly studied in the context of ERBB4, have for example been observed to regulate gene expression by acting as transcriptional coactivators or corepressors.

### ERBB4 ICD signaling in development and growth

Various processes in development and differentiation of different organs have been reported to be at least partially dependent on the functionality of nuclear ERBB4 ICD. The ICD has been shown to localize into the nuclei in mammary epithelial cells and found to induce differentiation of the mammary gland in a mouse model [61, 75, 76]. Maturation of mouse fetal lung type II epithelial cells has also been reported to be dependent on ERBB4 ICD formation in vitro [63, 77, 78]. In addition, it has been speculated that ERBB4 cleavage by gamma-secretase and production of ICD promotes mesenchymal cell proliferation during mouse heart valvulogenesis [79], although this assumption was not based on direct evidence of the activities of the cleaved ICD but interpretation of functional differences between cleavable and noncleavable ERBB4 isoforms [80, 81].

The molecular mechanisms relaying signaling from the nuclearly localized ERBB4 ICDs have reported to include nuclear interactions with several transcriptional activators or repressors. For example, it has been proposed that STAT5 is escorted to nucleus by ERBB4 ICD in mammary epithelial cells where the complex associates with beta-casein gene promoter inducing its activity and mammary differentiation [82]. During maturation of fetal lung in a mouse model, ERBB4 ICD has been reported to associate with transcription factors YAP, STAT5a and estrogen receptor beta [63, 77, 78]. Interaction of ERBB4 ICD with hypoxia-inducible factor 1 alpha (HIF-1α) in the nucleus results in stabilization of HIF-1α and increase in HIF-mediated transcription [76]. Interestingly, HIF-1α has been observed to regulate ERBB4 endocytosis and ERBB4-mediated differentiation of the mammary epithelial cells [83], indicating a reciprocal regulation by the two proteins. Association of estrogen-responsive gene expression and ERBB4 ICD has been identified, and ERBB4 ICD and estrogen receptor alpha localize as a complex to estrogen-inducible gene promoters, such as those regulating the expression of progesterone receptor and stromal cell-derived factor 1 (SDF-1). Supporting a functional role for soluble ICD, the localization was abolished when ERBB4 cleavage was blocked by a mutation preventing gamma-secretase cleavage [84]. Consistent with the biological role of these observations, *ERBB4* is expressed in estrogen receptor-positive breast cancers and ERBB4 ICD has been found to increase estrogen response element-mediated transcriptional activity [65]. The cleavage producing ERBB4 ICD may also promote proliferation or survival of estrogen-receptor-positive breast cancer cells. Indeed, overexpression of a cleavable ERBB4 isoform promotes breast cancer cell growth both in vitro [65, 73, 83, 85] and in vivo [86], and an antibody specifically blocking ERBB4 cleavage suppresses it [87].

Other transcriptional factors that have been reported to directly associate with and be modulated by ERBB4 ICD include ETO2, Krab-associated protein 1 (Kap1), and AP-2 [85, 88, 89].

### ERBB4 and RYK ICDs in neural development

ERBB4 and RYK ICDs have been associated with functions in neural development. In the developing mouse, the generation of ERBB4 ICD has been identified to be required for control of astrogenic differentiation [62]. The ERBB4 ICD was shown to interact with TAB2 and N-COR, an adaptor and a corepressor protein, respectively. These proteins are chaperoned to nucleus as a complex with the ICD and the complex binds to astrocytic-associated genes leading to repression of gene expression in vitro [62]. In addition, inhibition of gamma-secretase cleavage of ERBB4 has been indicated to prevent ligand-stimulated ICD formation and maturation of oligodendrocytes in vitro [90].

The cleavage of RYK has been reported to be required for Wnt3-dependent neuronal differentiation and nuclear localization of RYK ICD requires Wnt activation in neural progenitor cell cultures [31]. RYK ICD has been found to accumulate to nucleus during the cortical development, facilitating production of neurons from undifferentiated cells [31]. RYK ICD localization to nucleus has been identified to be dependent on SMEK1 and SMEK2 [91]. It was indicated that SMEK1 and SMEK2 chaperone the RYK ICD to nucleus and both SMEK and RYK ICD together associate with chromatin to *DLX1/2* intergenic regulation element and are involved in regulation of its transcription and neuronal differentiation of mouse primary cortical neural stem cells.

### Other RTK ICDs in the nucleus

Nuclear localization of PTK7 has been found to affect the proliferation of colon cancer cell lines [66]. This observation was based on overexpression of intracellular constructs in vitro, which may affect clinical interpretation of the findings.

Nuclear localization has also been observed for the ICD of TRKA by immunostaining human liver tissue samples with antibodies recognizing intracellular epitopes of TRKA, but not with antibodies recognizing the receptor ectodomain [68]. TRKA ICD was observed to colocalize with the splicing factor SC-35, associated with maturation of mRNA transcripts, and phosphorylation of the nuclear TRKA ICD in liver cells was interpreted as an indication of nuclear activity. These results led the authors to speculate that TRKA ICD could have a role in regulation of gene transcription and/or transcript processing. Research following the original observation has confirmed the cleavage of TRKA by gamma-secretase activity [17].

Stimulation with FGF1 has also been reported to induce gamma-secretase cleavage of FGFR3 in COS7 and T-Rex 293 cells. This was found to result in nuclear localization of the released ICD [30]. In contrast to a prevailing theme in gamma-secretase cleavage, shedding of FGFR3 was reported to require endocytosis. Also unlike the case of other RTKs, cathepsins rather than ADAM10 or ADAM17 were shown to be involved in the shedding of the FGFR3 ectodomain [30].

### Non-nuclear ICD signaling

In addition to promoting differentiation and growth, the ERBB4 ICD has been demonstrated to induce apoptosis in various breast cancer cell lines. In SKBr3 cells ERBB4 ICD has been found to localize into mitochondria and has been proposed to directly function as a proapoptotic protein, as the ICD contains a BH3 domain similar to the BH3-only type of BCL2 family members [70]. Consistently with this model, a point mutation in the transmembrane domain that was proposed to inhibit the gamma-secretase cleavage also suppressed the mitochondrial translocation of ICD and the associated induction of apoptosis [69, 70]. Moreover, ERBB4 ICD was observed to physically interact with BCL2, the prototype antiapoptotic protein, and overexpression of BCL2 was shown to reduce the ERBB4 ICD-induced cell death [70]. Furthermore, the ERBB4 ICD has been observed to phosphorylate MDM2 and promote its ubiquitination leading to increase in p53 and p21 levels [92], suggesting that there are also other mechanisms by which ERBB4 ICD may regulate apoptosis.

The cleavage of EPHB2 by gamma-secretase has been suggested to result in the formation of ICD residing in the cytoplasm where EPHB2 phosphorylates another receptor, *N*-methyl-d-aspartate receptor (NMDAR) in cultured rat primary neurons [72]. The phosphorylation is independent of SRC kinases normally associated with NMDAR signaling and promotes cell surface expression of NMDAR. The formation of EPHB2 ICD was proposed to potentially affect learning and memory through NMDAR [72].

Taken together, the soluble ICDs of RTKs have been observed to act as active signaling molecules with distinct functional properties. In the case of ERBB4 ICD, multiple functions have been reported in various cellular and organelle contexts. This growing body of evidence indicates that ICDs released from the cell membrane by gamma-secretase cleavage can have an active signaling role in various biological contexts.

## RTK downregulation by RIP

While ICDs released by gamma-secretase cleavage can add diversity to intracellular signaling by creating active signaling fragments, the cleavage also represents a mechanism of membrane receptor turn-over and degradation. The two steps of cleavage, generating the ectodomains and ICDs could participate together in downregulating RTK signaling through releasing the extracellular domain with the ligand-binding capacity to the extracellular space, and degradation of an active kinase domain, respectively. While there are a few examples indicating roles for RTK ectodomains in neutralizing the respective ligands (TRKB, AXL) [93, 94], the fate of extracellular fragments formed as a result of shedding is largely unknown. In this chapter, we will focus on describing examples of the RTK ICDs in providing negative regulation for RTK signaling.

### Rapid degradation of soluble RTK ICDs

As the ICDs released from the cell surface include the functional kinase domain, and a truncated ICD fragment anchored to the cell membrane prior to gamma-secretase cleavage may even possess enhanced kinase activity [95], the kinetics of ICD dephosphorylation and degradation are expected to play a central role in determining the functional consequence of ICD generation. Rapid degradation by proteasomes after gamma-secretase cleavage has been observed for at least with CSF1R, IGF1R, MET, and TIE1 [96–99].

CSF1R is quickly downregulated by gamma-secretase cleavage in macrophages during the activation of toll-like receptors by lipopolysaccharide, the major component of the cell wall of Gram-negative bacteria [37]. The resulting CSF1R depletion has been associated with macrophage activation. IGF1R expressed in HEK293 cells or murine embryonic fibroblasts has been reported to be constitutively shed followed by cleavage by the gamma-secretase complex resulting in formation of ICD that is rapidly degraded [97]. In epithelial cells, the soluble ICD of MET has been shown to be quickly degraded after constitutive gamma-secretase cleavage as well [98]. A noncleavable mutant full-length MET accumulates to membrane and displays ligand-independent signaling resulting in invasive growth of the cells [98], suggesting that RIP of MET is a mechanism for downtuning of signaling.

It has also been reported that proteolytic cleavage TIE1 in endothelial cells leading to ectodomain shedding and rapid ICD degradation may result in activation of TIE2 signaling [99]. As TIE1 cannot directly interact with the TIE2 ligands, angiopoietins, the mechanism may involve release of spatial hindrance within TIE1/TIE2 complexes or signaling executed by the ICDs. These findings provide another interesting example of how downregulation of one RTK by RIP can result in increased activity of another RTK.

### Inhibition of VEGFR signaling by RIP

A few articles have addressed the role of RIP in regulating signaling of the VEGFR subfamily of RTKs. Pigment epithelium-derived factor (PEDF) has been reported to induce shedding and gamma-secretase cleavage of VEGFR1 resulting in inhibition of VEGF-induced endothelial tube formation [71] and endothelial cell permeability [33]. Induction of VEGFR1 ICD formation in retinal microvascular endothelial cells was described to be associated with reduction of VEGFR1 phosphorylation [33]. As VEGFR1 is largely considered a negative regulator of angiogenesis [100], the results could also be explained by gamma-secretase-cleavage-associated release of the VEGFR1 ectodomain and sequestration of ligands such as VEGFA that also activate the proangiogenic VEGFR2. This latter conclusion was supported by analyses of VEGFR1 cleavage in leukemic cells representing the few nonendothelial cell types naturally expressing VEGFR1 [34].

In addition to VEGFR1, VEGFR2 has been reported to be cleaved by gamma-secretase in retinal pigment epithelium cells following induction by PEDF, a VEGFR1 agonist, leading to reduced VEGFR2 agonist-induced cellular permeability [101]. In this model, the effect of PEGF was shown to require indirect gamma-secretase-mediated processing of VEGFR2, but not of VEGFR1. These results provide an illustrative example of how the gamma-secretase cleavage of the two VEGFRs, VEGFR1 and VEGFR2, is differently regulated depending on the cellular context.

The third member of the VEGFR subfamily of RTKs, VEGFR3, was also found to be cleaved in a systematic screen for gamma-secretase-sensitive RTKs [17]. While inhibition of gamma-secretase activity reduced VEGFR3-induced growth when the receptor was overexpressed in fibroblasts [17], no biological contexts for signaling of soluble VEGFR3 ICD has to date been reported.

## Therapeutic implications in cancer

### Potential of RTK cleavage as target for treatment

RTK signaling is frequently altered during carcinogenesis, and tumor cells may become addicted to specific activating variations in RTK sequence or copy number [102]. Therefore, modulation of RTK signaling by gamma-secretase could affect processes relevant for tumor growth. It is of note that the increased RTK activity can also result from inadequate downregulation as well [103]. Thus, any defects in the components of shedding and gamma-secretase cleavage machinery or in the regulation of cleavage process can in principle regulate tumor formation.

Most of the human RTKs are targeted by one or several of the currently approved cancer drugs. These drugs belong to two major drug classes: Tyrosine kinase inhibitors that block RTK signaling by competing for ATP to block the intrinsic RTK kinase activity, or monoclonal antibodies that block ligand interaction or RTK dimerization, facilitate RTK endocytosis and degradation, or augment antibody-dependent immunological mechanisms [104, 105]. Since signaling by several of these RTKs is also modulated by gamma-secretase activity, it is plausible that regulation of RTK processing by modulators of gamma-secretase cleavage could also affect the tumor promoting activity of the RTKs. Indeed, chemical gamma-secretase inhibitors have demonstrated antitumor activity in tumor models and multiple clinical trials have been carried out for gamma-secretase inhibitors [106]. Most of the activities of these inhibitors, however, have been attributed to their ability to block notch signaling and there is very little information available about the role of RTKs in mediating either the antitumor activity or adverse effects of the gamma-secretase inhibitors [34, 106]. The clinically tested gamma-secretase inhibitors have demonstrated clinical antitumor effect, but the adverse effects of the first-generation inhibitors have not been tolerable. The increasing number of gamma-secretase substrates introduces a challenge for developing inhibitors selective only for the desired target. In addition, adverse effects of gamma-secretase modulators may also result from induction of cleavage of gamma-secretase substrates by other membrane proteases such as rhomboid, which normally would not cleave these proteins [107].

### Role of RTK cleavage in resistance to RTK-targeted therapies

While the relevance of RTK cleavage as a resistance mechanism has not been extensively studied, one can speculate on several possible mechanisms by which RTK processing could affect the sensitivity of cells to currently existing RTK-targeted therapies. Indeed, the fact that gamma-secretase-mediated RTK cleavage releases receptors from the membrane alters the well-characterized RTK signaling mode that has also been the primary target for drug development.

The release of ectodomain by shedding does not only deplete the cells from structures available for interaction with therapeutic antibodies binding to the ectodomains but can also create a decoy receptor that binds the antibody within the extracellular space further neutralizing the effect on cells that would still express an intact receptor. Small molecule inhibitors against RTK kinase domains, on the other hand, could still be active therapeutic agents even after the release of a soluble ICD domain into intracellular compartments. However, in spite of attempts to develop ADAM inhibitors as cancer drugs [35], recent evidence indicates that suppressing RTK shedding may not be desirable, as this reduces the production of soluble decoy receptors, and stabilizes fully active RTKs at cell surface. An analysis of the effects of MAPK pathway inhibitors on the shedding of RTKs indicated that reduced shedding of RTKs, such as AXL, MET, and ERBB4, was associated with resistance to MEK inhibitors in patients with melanoma [108]. The reduced RTK shedding was associated with inhibition of ADAM10 by increase of cell surface TIMP-1. Similarly, in colorectal cancer with KRAS mutations, the reduced shedding of MET by ADAM17 has been observed to create resistance to MEK inhibitors [109].

## Future aspects

Most of the cleavable RTKs have no identified cellular function for their gamma-secretase released ICDs, and only a handful of RTK ICDs have any identified function. Future research will need to expand our view of ICD signaling beyond current understanding that is to a large extent based on reports focusing on one RTK, ERBB4. As most of our understanding about the biological relevance of gamma-secretase-mediated processing of RTKs is based on in vitro observations, there is also need for more thorough in vivo validation of the findings. This goal would greatly benefit from the characterization of cleavage sites and regulatory mechanisms critical for the process, thereby enabling specific molecular and genetic interference with the events.

Development of new and more specific inhibitors targeting RTK cleavage needs better understanding of the biological significance, as well as about the mechanisms by which gamma-secretase substrates are distinguished from nonsubstrates. In addition, almost all the knowledge about molecular mechanisms and signaling events associated with regulation and substrate recognition by gamma-secretase is based on the research focusing on APP cleavage and, to some extent, on notch cleavage as well. Validation of these findings among RTKs would provide broader spectrum of tools for development of therapeutic agents specifically interfering with gamma-secretase-mediated cleavage of RTKs.
